# Long‐distance swimming by African lions in Uganda

**DOI:** 10.1002/ece3.11597

**Published:** 2024-07-10

**Authors:** A. Braczkowski, L. Ochse, B. Atukwatse, O. Cornille, C. O'Bryan, P. Lindsey, R. Kotze, L. Gibson, D. Biggs

**Affiliations:** ^1^ Centre for Planetary Health and Food Security, School of Environment and Science Griffith University Nathan Queensland Australia; ^2^ Department of Conservation Management Nelson Mandela University George Western Cape South Africa; ^3^ Volcanoes Safaris Partnership Trust, Kyambura Lion Project Kampala Uganda; ^4^ School of Environmental Science and Engineering Southern University of Science and Technology Shenzhen China; ^5^ Rolling Label, Le Petit Provence Estate Franschhoek South Africa; ^6^ System Earth Science Maastricht University Venlo The Netherlands; ^7^ Wildlife Conservation Network San Francisco California USA; ^8^ Wildlife Conservation Research Unit University of Oxford Oxfordshire UK; ^9^ School of Earth and Sustainability Northern Arizona University Flagstaff Arizona USA; ^10^ Centre for Complex Systems in Transition, School of Public Leadership Stellenbosch University Stellenbosch South Africa

**Keywords:** African lionUganda, Albertine rift, carnivore, felid, Panthera leo, Queen Elizabeth National Park, swimming

## Abstract

Earth's most imperiled and iconic wildlife are facing tough decisions under increasing human pressure and limited resources. Swimming across rivers and water bodies filled with high densities of predators may be one such example. In African lions *Panthera leo*, previous water crossings (recorded in the peer‐reviewed and gray literature, on film, and found using Google Search, and YouTube) have recorded distances ranging from <10 to 100 m, with some resulting in mortality by Nile Crocodiles *Crocodylis niloticus*. However, we observed a coalition of male lions swimming >1 km across Uganda's Kazinga channel located in the Queen Elizabeth National Park six times, and recorded this behavior on film on February 1st 2024. We speculate that three factors could be driving these lions to take long‐distance swims with a high density of crocodiles and hippos *Hippopotamus amphibius*, namely (1) the lack of lionesses in this ecosystem, (2) intraspecific fights over territory with other male coalitions, and (3) the only other land connection giving lions access to the peninsula is a small road bridge with a strong human presence.

## Graphical abstract

We present a visual documentation of long‐distance swimming by African lions in Uganda. This first recorded visual evidence shows lions in the Queen Elizabeth National Park are engaging in risky behavior to find females.

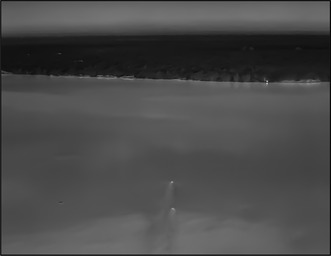

## 1 INTRODUCTION

Large charismatic mammals are facing challenges to their movements in an increasingly human dominated world (Campos‐Arceiz et al., 2022). According to a recent evaluation by the UN (UNEP‐WCMC, 2024), this anthropogenic pressure, particularly in the form of habitat loss and fragmentation, is driving the decline of 44% of the world's migratory species. Long‐distance dispersal events (Hussain et al., 2022), temporary refuges in human infrastructure (Odden et al., 2014), and even persistence in war zones (Daskin & Pringle, 2018) have become common in large felids (Fattebert et al., 2013), elephants (Fernando et al., 2023), and bears (Bartoń et al., 2019). Such risk taking is common across both sexes in the animal kingdom, but due to antagonistic interactions, and polygamy, some species die younger in their search for females, and attempts to breed (Lemaître et al., 2020). In African lions, males already pay a high cost for large, impressive, sexual signals (e.g. manes, Lemaître et al., 2020) and take tremendous risks to find mates, establish tenure and defend lionesses and their cubs (Packer, 2023; Packer & Pusey, 1997), and this is likely compounded when there is limited access to females or significant human pressure. Indeed, anthropogenic or natural barriers may impede movements, population connectivity (see Cozzi et al., 2013), and access to mates. Rivers, channels, and lakes are examples of significant natural barriers to lion movements likely due to the presence of hazardous species such as crocodiles (*Crocodylus niloticus*) and hippos (*Hippopotamus amphibious*, see Supporting Information S1). Lions are known to hunt both crocodiles and hippos on occasion, but when in water they themselves become vulnerable to depredation from crocodiles (a species that can weigh up to 4× more than a large male lion, see Supporting Information S1 for a collection of video evidence documenting Nile crocodile attacks on African lions) or aggression from hippos, with limited ability to escape. However, information is scant regarding their ability to swim, and specifically swim long distances. To overcome this gap, we present evidence for six such events that took place over a year in western Uganda. We then compile available evidence of this behavior by the species from a variety of peer‐reviewed and gray sources, and discuss this in the context of other large felids and implications for their conservation.

## 2 OBSERVATIONS

**FIGURE 1:**
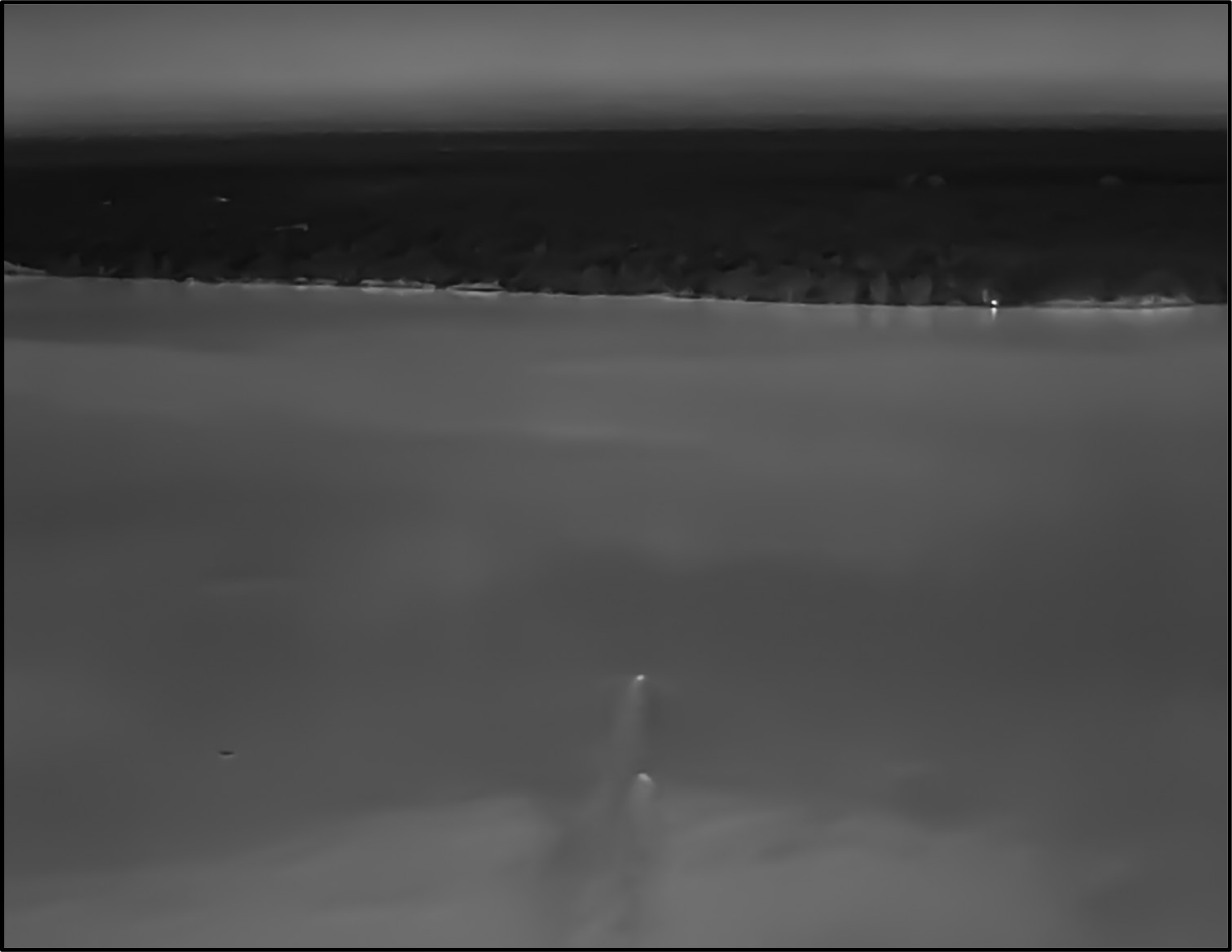
The heat signatures of two male African lions (*Panthera leo*) swimming towards the Katunguru peninsula of Queen Elizabeth National Park on February 1st, 2024. The swim distance is estimated to be between 1 and 1.5 km, and is the first visually documented long‐distance swimming event recorded for the species. Image taken by the second author, Luke Ochse, using a DJI Matrice 300 drone fitted with a H20T thermal camera.

**FIGURE 2:**
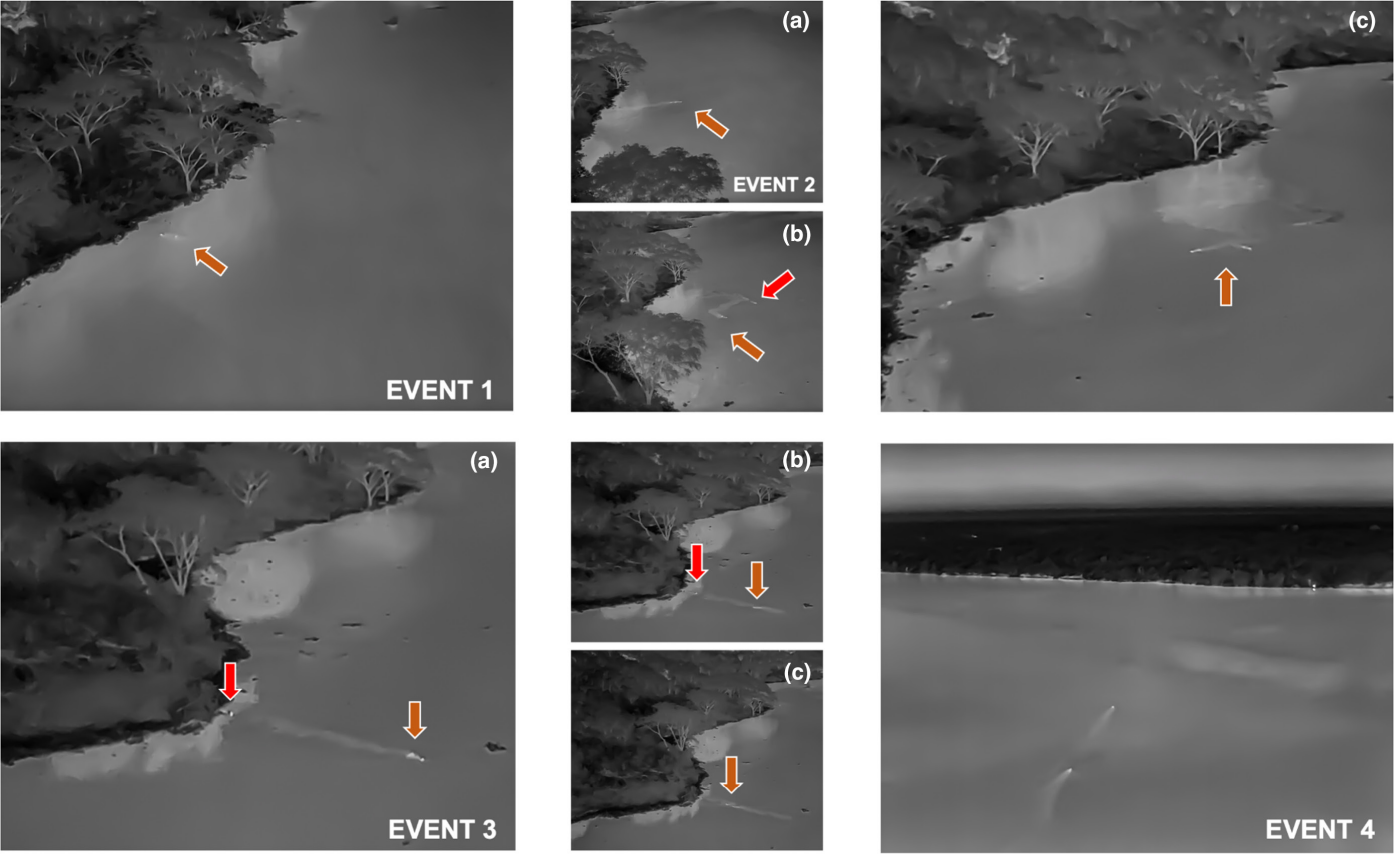
On 1st February at 21:13 the male coalition begins to move along the waters edge, entering water for the first time (Event 1) but quickly turn back after <15 m. They then attempt a second crossing traversing roughly ~80 m before again turning to shore (EVENT 2a), and then splitting up (EVENT 2c), after what appears to be a large disturbance in the water (likely a hippo or large crocodile—based on the large heat signal). One male attempts the crossing for a third time but after swimming ~80 m it appears that something frightens him (EVENT 3a—possibly an animal or current in the channel), he turns back towards shore and his coalition mate appears to swim out to either aid or join him (EVENT 3b). Both males make their final swim of ~1.3 km shortly thereafter and we finished our observation roughly 750 m from the initial entry point (EVENT 4) with both males in the water on route to the Katunguru region of the park. We obtained a visual of the male coalition on Sunday 4 February, roughly 80 m from a gorge where we suspect the males entered. The entire sequence of events is presented in Video 1.

We captured night time footage of a coalition of two male lions (local study names used by several lion projects and the Uganda Wildlife Authority: Jacob, a three legged lion who lost his foot in a poachers trap, and his brother Tibu). The two males were filmed swimming across the Kazinga channel, a waterway connecting two lakes (George and Edward). The footage was captured on 1 February 2024 with a DJI Matrice 300 drone carrying a H20T thermal camera payload. We followed the approach of Pollock et al. (2022) and Brunton et al. (2019), maintaining an altitude distance of ~50–75 m away from the lions. The pair are seen attempting to cross 3 times (Figure 1) but returned back to shore shortly after entering the water each time (due to what appears to be an animal trailing the lions, possibly a hippopotamus or Nile crocodile, but identity cannot be confirmed, Figure 2) before setting off on the >1 km crossing (Video 1). The long‐distance swim was successful and our team recorded a visual observation of the two males roughly 4 km from the shoreline of their estimated exit point on Sunday 4 February 2024. We estimate the linear distance to be approximately 1.1 km from the initial swim commencement, and closer to 1.5 km once the directionality changes observed during the swim via thermal drone were factored in. The swim was likely a consequence of two intraspecific fights with two rival coalitions (fought and chased on the eve of the 28th January 2024—00:03 AM, and chased by another coalition on eve of 30th January—21:19 PM). We hypothesize that the broader reasoning for the swim from the Kazinga village region (the area where the coalition spends the majority of its time) on the 4 October 2023 (the initial swim that led to the males ranging in the channel track region of Mweya) is a lack of lionesses in the region, and the skewed sex ratio observed in the Queen Elizabeth National Park lion population (Braczkowski, Gopalaswamy, Nsubuga, et al., 2020), while the return swim we filmed back to the Kazinga region is likely the result of intraspecific fighting over territory. Indeed, a significant motivator of African male lion movements is the presence and distribution of lionesses and their prides (Packer, 2023). In 6 months of intensive monitoring and filming of the coalition we only observed one female with the coalition for a period of 3 days. The highest density of lionesses and male lions that have tenure over their prides is in the Mweya‐Kasenyi region where these males swam to. These include the Kaine coalition of six adult male lions (~5–6 years of age), a two male coalition (Chuma and Sidi), and another two male coalition (Mike and Kari). These three coalitions have tenure over seven adult lionesses. Our field team observed evidence for this swimming behavior on six other occasions: 1 February 2023, 8 June 2023, 29 June 2023, 17 August 2023, only one (Tibu) on 20 August 2023, 4 October 2023, and 29 February 2024. Video 2 shows the lions moving towards the water preceeding the swim attempts.

**VIDEO 1:** Footage of the two male coalition (study names Jacob and Tibu) making the three crossing attempts before embarking on the final swim to the Kazinga village region of the national park.

**VIDEO 2:** Video showing the two male lions (Jacob and Tibu) moving towards the Kazinga channel before embarking on the swims.

## 3 LONG‐DISTANCE SWIMMING IN LIONS AND OTHER BIG CATS

Swimming behavior in large felids is common knowledge among experts, but to our understanding no work has documented long‐distance swimming in African lions. Following our field observations of African lions swimming long distances in Uganda, we performed an online search for literature and public observations of swimming events to determine if long‐distance swimming has been recorded previously. We conducted our search using Google Scholar, Web of Knowledge, media articles on Google, and video material on YouTube using two keyword combinations: “African lion” and “swim”, and “Panthera leo” and “swim” (Supporting Information S2). We limited our search to the first 45 pages of Web of Knowledge and first 10 pages of Google Scholar (Braczkowski, Gopalaswamy, Elliot, et al., 2020), the first 50 videos on YouTube, and 10 scrolls in Google Search.

Our search produced a myriad of observations of swimming behavior of African lions and other big cats (see Supporting Information S1). Specifically, we found one peer‐reviewed article by Inogwabini and Inogwabini (2020) who hypothesize that lions could be crossing the Kwa‐Kasaii and Congo rivers (a distance of 200 m ‐ ≥ 5 km wide in some parts), but present no visual evidence, nor any formal or even anecdotal evidence for this. We also found four videos on YouTube of African lions displaying swimming behavior mainly in the swamps of the Okavango Delta (see Supporting Information S1). All of these were short swims of less than 50 m in length. We also found four articles in the gray literature showing lions moving across rivers, most notably an adult male called “Dynamite” crossing the 100 m‐wide Zambezi from northern Zimbabwe to Livingstone, Zambia on January 15th 2012 (Roberts 2012). Another notable event took place in November 2023 where a sub‐adult male crossed the Rufiji River bordering the north of Tanzania's Selous Game Reserve. This crossing was estimated at between 90 and 300 m (see Lion Landscapes; http://tinyurl.com/2zn396b2). The closest distance to our observation was recorded in other felid species, such as that by Stratton et al. (2022) of a dispersing male puma *Puma concolor* that swam 1.1 km from the Olympic Peninsula to Squaxin Island in Puget Sound, Washington, USA. Another example is video evidence of a male tiger *Panthera tigris* crossing the Brahmaputra river in northeastern India where he can be seen open water swimming in the river, which in some parts reaches >1 km in width (see: https://www.hindustantimes.com/cities/others/royal‐bengal‐tiger‐that‐swam‐across‐brahmaputra‐rescued‐sent‐to‐guwahati‐zoo‐101671596536772‐amp.html). Similarly Miththapala et al. (1991) noted tigers crossing the wide tidal rivers at the mouth of the Ganges in the Sunderbans. Jaguars (*Panthera onca*) are well known for their swimming ability in wetlands like the Pantanal and in floodplain forests in Brazil and are known to hunt caiman (*Caiman yacare*) from both inside and outside the water (Azevedo & Verdade, 2012; Rabelo et al., 2019). However, as caiman are much smaller that their African cousins, the Nile crocodile, and will likely avoid swimming jaguars for fear of predation, caiman pose little threat to jaguars crossing channels and rivers. These crossings are also likely to be over short distances between islands (Rabelo et al., 2019). As such, big cats are documented to swim long distances, but the data are scarce and inconsistent.

## 4 CONSERVATION IMPLICATIONS

Many questions arise from our observation of long‐distance swimming by African lions in Western Uganda. Given the increasing human pressure that has led to local declines in lion populations, we suggest that this behavior could be signaling a lack of terrestrial access to resources or potential mates. Male lions in particular are known to be less risk averse when traveling through novel landscapes, particularly males of dispersal age (Elliot et al., 2014), or males that have been displaced by new males, in search of new prides to further their reproductive outputs (Packer, 2023). This is supported by the fact that most of the recorded long‐distance swims we have come across in *Panthera* and *Puma* species have been undertaken by adult or sub‐adult males. We argue that the most plausible hypothesis is the very low number of lionesses in this system. We make this assertion based on the intensive observations of this male coalition, only once during 2023 did we witness them with a lioness on the Kazinga‐Katunguru side of the channel. All other observations with or within close proximity to females were made on the northern size ie. Mweya side of the park—which has the highest concentration of lions (Braczkowski, Gopalaswamy, Nsubuga, et al., 2020).

While lions are known to live close to humans across some parts of their range (see for example the regular proximity to human settlements described in Banerjee et al., 2013), an alternative explanation, although a less plausible one, could be that the coalition is taking greater risk to avoid human contact on the small connecting bridge between the northern and southern parts of the Kazinga chanel. The only terrestrial crossing of the Kazinga Channel is a short bridge (~50 m in length) located immediately after Katunguru village and is guarded by two armed guards (heavy gunners from the Uganda Peoples Defense Force). In the Okavango Delta, lions and leopards (*Panthera pardus*) regularly make use of bridges where available (R. Kotze pers. Observation), indicating that lions perceive this as less risky than crossing channels. While lions are known to mediate risk associated with humans through spatial or temporal avoidance (Loveridge et al., 2017; Oriol‐Cotterill et al., 2015), sometimes, the selection of alternative routes or habitats can be maladaptive or risky in themselves (Battin, 2004; Loveridge et al., 2017). River crossings in Africa come with considerable risk of injury, or even death, from encounters with the much larger Nile crocodile or hippopotamus, as evidenced from the several video observations in Supporting Information S1. Increased risk taking in the absence of alternative routes as a result of human pressure (including through the persecution of lionesses, Braczkowski, Gopalaswamy, Nsubuga, et al., 2020) could thus lead to increased mortalities.

Positively, records like this indicate that where motivation is sufficient, lions can cross large rivers, which are commonly perceived as barriers to connectivity between extant lion populations (Cozzi et al., 2013). This has implications for conservation corridor planning between isolated populations. We urge future research to explore these long‐distance swimming behaviors and functional habitat connectivity of big cats in human‐dominated landscapes.

## AUTHOR CONTRIBUTIONS

**A. Braczkowski:** Conceptualization (lead); data curation (lead); formal analysis (lead); funding acquisition (supporting); investigation (lead); methodology (lead); project administration (supporting); resources (lead); software (lead); supervision (lead); validation (lead); visualization (lead); writing – original draft (lead); writing – review and editing (lead). **L. Ochse:** Investigation (supporting); methodology (supporting); writing – review and editing (supporting). **B. Atukwatse:** Investigation (supporting); project administration (supporting); writing – original draft (supporting); writing – review and editing (supporting). **O. Cornille:** Investigation (supporting); project administration (supporting); writing – original draft (supporting); writing – review and editing (supporting). **C. O'Bryan:** Conceptualization (supporting); writing – original draft (supporting); writing – review and editing (supporting). **P. Lindsey:** Writing – original draft (supporting); writing – review and editing (supporting). **R. Kotze:** Validation (supporting); writing – original draft (supporting); writing – review and editing (supporting). **L. Gibson:** Funding acquisition (supporting); writing – original draft (supporting); writing – review and editing (supporting). **D. Biggs:** Funding acquisition (lead); project administration (supporting); writing – original draft (supporting); writing – review and editing (supporting).

## FUNDING INFORMATION

Funding for this work was provided from multiple sources: Griffith University's Centre for Planetary Health and Food Security, Griffith University's Media and External Communications centre, Northern Arizona University, and the Southern University of Science and Technology. The open access charges for this article were supported by Luke Gibson and the School of Environmental Science and Engineering, Southern University of Science and Technology, Shenzhen, China.

## CONFLICT OF INTEREST STATEMENT

The authors declare that there are no conflicts of interest.

## Supporting information


Data S1

Data S2

Data S3

## Data Availability

The raw results from our literature review are provided in Supporting Information S2. The footage of the lions crossing the Kazinga channel is provided freely in Video 1.
