# Supplementary material for: Long‐distance swimming by African lions in Uganda
Source: Ecol Evol. 2024 Jul 10;14(7):e11597. doi: 10.1002/ece3.11597 (PMC11236087; doi:10.1002/ece3.11597)
Supplement: Supplementary file 1 — Data S1 [file ECE3-14-e11597-s003.docx]

**Supporting Information 1**

***Results from YouTube Search - “African lion: swim”***

<https://www.youtube.com/watch?v=3NbgK9EaKyk> - lions swimming in Okavango Delta

[https://www.youtube.com/watc h?v=XabZLTpugN8](https://www.youtube.com/watch?v=XabZLTpugN8) - lions crossing short river swamp in Okavango Delta

<https://www.youtube.com/watch?v=5HUJKILrVlw&t=36s> - lions attacked by crocodile while crossing small river (Kings Pool camp, Botswana)

<https://www.youtube.com/watch?v=aWM2gywc0Ew&t=552s> - documentary showing lions in the water in Okavango Delta (no specific footage on crossing large water bodies)

<https://www.youtube.com/watch?v=Qn_AP9liBKw> - three lions getting attacked by a hippo in Selinda reserve, Botswana.

<https://www.youtube.com/watch?v=CEBiym5iQ08> - two male lions in a small dam (not swimming interacting)

<https://www.youtube.com/watch?v=yM66WpYEPVE> - footage of lions swimming across a small water body in the Okavango delta

***Results from YouTube Search - “Panthera leo: swim”***

<https://www.youtube.com/watch?v=OoAmGUpvRyc> - footage of a male lion coalition crossing the Ingwelala, South Africa (appears to be <50 metres in width)

<https://www.youtube.com/watch?v=5F_h4W9pj34> - three male lion coalition swimming across a ~30 metre channel in Okavango Delta

<https://www.youtube.com/shorts/sxKIhk6Cwls> - lion cub swimming across small river to its mother

<https://www.youtube.com/shorts/amhdBCx3IV4> - lion hunting in water

<https://www.youtube.com/shorts/yG1mDtku7wA> - male lion attacked by hippo in water

***Evidence of crocodiles attacking African Lions***

Male lions engaged by Nile crocodile - one croc seemingly kills one male: <https://www.youtube.com/watch?v=5HUJKILrVlw>

Male lion attacked by crocodile: <https://www.youtube.com/shorts/mGyK4t4QCZA>

<https://onlinelibrary.wiley.com/doi/10.1002/ece3.11016>

***Evidence of hippos attacking African Lions***

Three male lions being attacked in a river by a hippopotamus: <https://www.youtube.com/watch?v=Qn_AP9liBKw>

Male lion being attacked by a hippo in a river: <https://www.youtube.com/watch?v=29vIeTcabQ8>

**Detailed Georeferenced Description of Swim Crossing Event**

On 1st February 21:13 males begin to move along waters edge (-0.143416, 30.003296), entering water for the first time at 21:28. They then attempt their first crossing at 21:40 but turn back after <15 m (EVENT 1). They then attempt their second crossing at 21:47 traversing roughly ~80 m before again turning to shore (EVENT 2 A), and then splitting up (EVENT 2 C), after what appears to be a large disturbance in the water (likely a hippo or large crocodile - based on the large heat signal). Both lions return to shore at 21:50 PM. Lions again move at 21:51 PM, momentarily rest for ~ 12 minutes till 22:03 when they again enter the water and wait. At 22:10 PM one male attempts the crossing for a third time but after swimming ~80 m it appears that something frightens him (EVENT 3 A), he turns back towards shore and his coalition mate appears to swim out to either aid or join him (EVENT 3 B), they reach shore at 22:12 PM. At 22:13 PM both males make their final swim of ~1.3 km (final entry point was at -0.145659, 30.002792) we finished our observation roughly 750 m from the initial entry point (EVENT 4) with both males in the water on route to the Katunguru region of the park (22:25 PM). We obtained a visual of the male coalition on Sunday 4 February -0.141207, 30.040199, roughly 80 meters from a gorge where we suspect the males entered. The entire sequence of events is presented in Video 1.
