# Supplementary material for: Long‐distance swimming by African lions in Uganda
Source: Ecol Evol. 2024 Jul 10;14(7):e11597. doi: 10.1002/ece3.11597 (PMC11236087; doi:10.1002/ece3.11597)
Supplement: Supplementary file 2 — Data S2 [file ECE3-14-e11597-s002.pdf]

Supporting Information 2

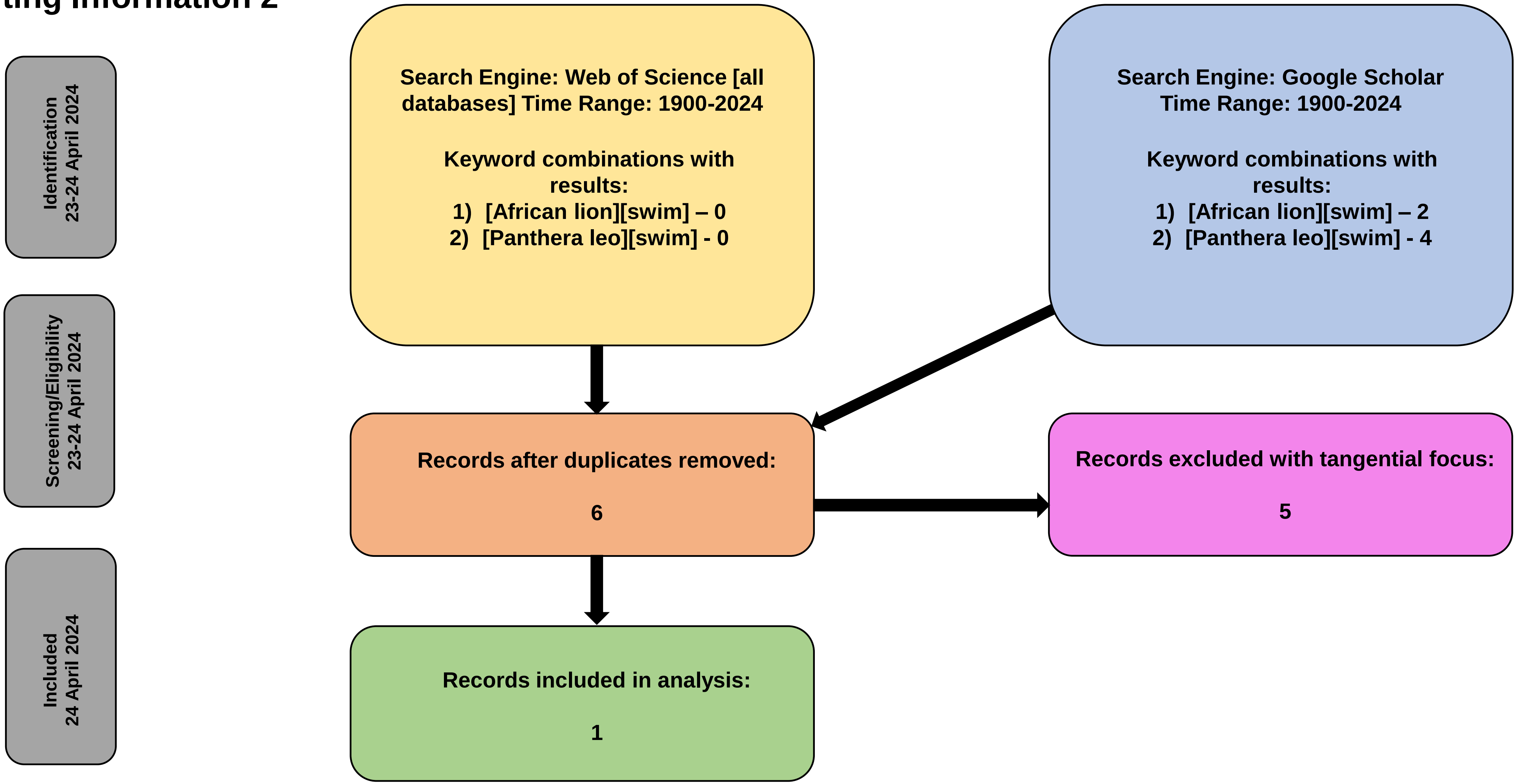

Supporting Figure 1: An adapted PRISMA flow diagram summarizing the identification, screening and inclusion of scientific articles found on Web of Science (WOS) and Google Scholar. Two records were excluded as they did not explicitly discuss African lions swimming. The period for the search for both Web of Science and Google Scholar was 1900-2024 (23 April 2024). We limited our search to the first 45 pages of WOS and the first 10 pages of Google Scholar. We also searched the first 50 videos on YouTube, and ten scrolls in Google Search using the same keywords as those in our WOS and Google Scholar.

Table 1: Peer-reviewed, grey literature, and video search of African lion swimming behaviour. None of our findings can confirm long distance events > 1 km in length.

Keyword combination	Source Searched	Date of Search	Date Range of Material Searched	Potential Papers/Articles/Videos Recovered	Final Materials Relevant to Study
“African lion” and “swim”	Web of Science	21st Feb 2024	1900-2024	0	0
“Panthera leo” and “swim”	Web of Science	21st Feb 2024	1900-2024	0	0
“African lion” and “swim”	Google Scholar	21st Feb 2024	1900-2024	2	0
“Panthera leo” and “swim”	Google Scholar	21st Feb 2024	1900-2024	4	1
“African lion” and “swim”	YouTube	21st Feb 2024	Sorted by Relevance*	7	7
“Panthera leo” and “swim”	YouTube	21st Feb 2024	Sorted by Relevance*	5	5
“African lion” and “swim”	Google Search	21st Feb 2024	Any Duration, Any Time	0	0
“Panthera leo” and “swim”	Google Search	21st Feb 2024	Any Duration, Any Time	0	0
